# Aggressive vertebral hemangiomas contain no adipose tissue resulting in thoracic spine kyphosis: A case report

**DOI:** 10.1097/MD.0000000000037885

**Published:** 2024-04-19

**Authors:** Liang-Jie Wang, Hong-Mei Zou, Feng Hou, Guan-Xi Wang, Chuan-Ping Gao

**Affiliations:** a Department of Radiology, The Affiliated Hospital of Qingdao University, Qingdao, China; b Department of Radiology, The Affiliated Qingdao Third People's Hospital, Qingdao University, Qingdao, China; c Department of Pathology, The Affiliated Hospital of Qingdao University, Qingdao, China; d Department of Radiology, Songshan Hospital of Qingdao University Medical College University, Qingdao, China.

**Keywords:** aggressive vertebral hemangioma, case reports, kyphosis, spine

## Abstract

**Rationale:**

Aggressive vertebral hemangiomas (AVHs) destroy continuous vertebral bodies and intervertebral discs and resulting in spinal kyphosis is extremely rare. The very aggressive behavior was attributable to its significant vascular component and contained no adipose tissue.

**Patient concerns:**

We report a case of thoracic spine kyphosis of AVHs with multiple vertebral bodies and intervertebral disc destruction in a 45-year-old woman.

**Diagnoses:**

Based on the imaging studies, the patient underwent surgical removal of this lesion and spinal reconstruction. Histopathology consistent with vertebral hemangioma and contained no adipose.

**Interventions:**

The patient underwent surgical removal of the lesion and spinal reconstruction. After subperiosteal dissection of the paraspinal muscles and exposure of the laminae, the laminae of the T5–7 vertebrae were removed and exposing the lesion. The lesion was soft and showed cystic changes, completely curetted and autogenous bone was implanted. Vertebroplasty was performed through T3-T9 pedicles bilaterally. Pedicle screw fixation was performed for segmental fixation and fusion.

**Outcomes:**

After 9 days of operation, the incision healed cleanly and free of pain. She was discharged in good general condition. The patient remained asymptomatic after follow-up 6 months of postoperative.

**Lessons:**

AVHs destroy multiple vertebral bodies and intervertebral discs and resulting in spinal kyphosis is extremely rare.

## 1. Introduction

Vertebral hemangiomas (VHs) are extremely common, their incidence being between 10% and 27% in adults.^[1]^ They are characterized by proliferation of blood vessels and may become aggressive by invading the spinal canal and/or paravertebral space,^[2]^ thereby causing cord compression and neurological symptoms. Aggressive vertebral hemangiomas (AVHs) can cause extensive osteolytic vertebral bone destruction without a honeycomb appearance and/or the “polka-dot sign” characteristic of classic VHs. According to 1 previous study, aggressive behavior is related to their fat content: Hoyle et al reported that lipid-poor lesions do not behave aggressively.^[3]^ However, Laredo et al^[4]^ reported 3 compressive VHs evaluated by magnetic resonance imaging (MRI) that showed low signal intensity on T1WI images. Here, we describe a case of a 45-year-old woman with an AVH that caused osteolytic destruction of the T5–7 vertebral bodies and T5–6 and T6–7 intervertebral discs, resulting in thoracic kyphosis. Pathological examination of the operative specimen revealed that the lesion comprised predominantly of blood vessels and contained no adipose tissue. To the best of our knowledge, this is the first reported case of an aggressive spinal hemangioma containing no adipose tissue and having destroyed multiple vertebral bodies and interverbal discs, which produced a single cavity and caused kyphosis.

## 2. Case report

**Figure 1.**
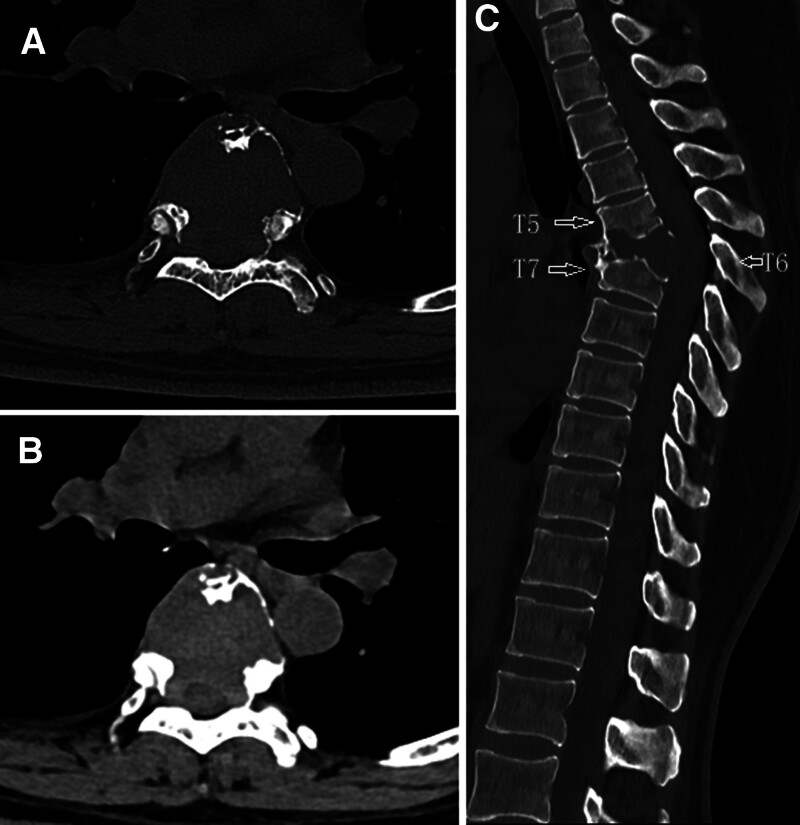
CT axial bone window (A) and soft tissue window (B) showing vertebral body bony destruction. Sagittal reconstruction (C) showing that the T5–7 vertebral bodies and intervertebral discs have been destroyed, causing thoracic kyphosis. CT = computed tomography.

**Figure 2.**
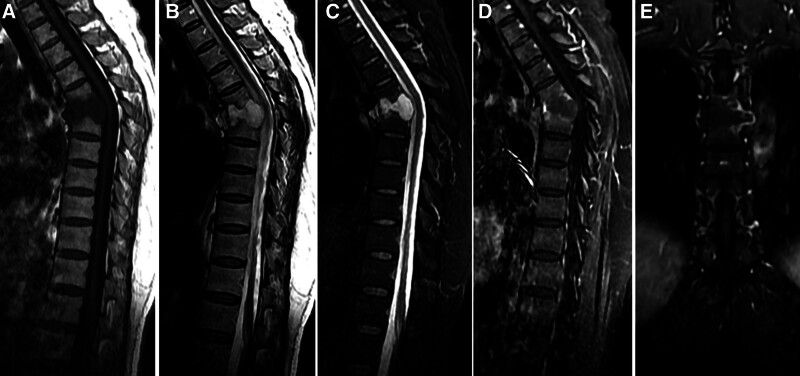
CT images of the lesion showing homogenous hypointensity on T1WI (A) and hyperintensity on T2WI (B) and STIR sequences (C). Post-contrast images show circumferential enhancement of the lesion (D, E). CT = computed tomography, STIR = short T1 inversion recovery.

The patient, a 45-year-old woman, a spinal lesion was found incidentally on computed tomography (CT) of her thoracic spine. On examination, no neurological abnormalities were found. Axial CT images showed osteolytic destruction of several thoracic vertebral bodies (Fig. 1A). The osteolytic area was densely homogeneous, the CT value being approximately 61 HU (Fig. 1B). Sagittal CT reconstruction images showed a single area of bone destruction on the inferior aspect of the T5 vertebral body, the whole T6 vertebral body, and superior aspect of the T7 vertebral body. The margins of the resultant cavity were well-defined and partially sclerotic. The intervertebral discs between T5–6 and T6–7 were completely destroyed. She had kyphosis at the level of T6. The spinous process of T6 was intact (Fig. 1C, right-hand arrow). The lesion appeared as a homogeneous hypointense lesion on T1WI, whereas high signal intensity was seen on T2WI and short T1 inversion recovery (Fig. 2A–C). The lesion showed circumferential enhancement on post-contrast MRI (Fig. 2D–E).

Because of her thoracic kyphosis, the patient was referred for surgery.

After successful induction of general anesthesia, the patient was positioned prone, and the skin sterilized with 2.5% povidone-iodine. An approximately 30-cm, straight, midline incision was made from T3 to T9, incising the epidermis, dermis, and subcutaneous tissue. The paravertebral muscles were then retracted as far as the outer edge of the facet joints and pedicle screws inserted into the pedicles of T3-T9 bilaterally. After using C-arm fluoroscopy to check that the position, depth, and angle of the pedicle screws were correct, the spinous processes of the T3–9 vertebrae and the laminae of the T5–7 vertebrae were removed, exposing the thecal sac. The lesion was soft and showed cystic changes. Tissue samples were excised and sent to pathology. Examination of frozen sections revealed abundant proliferating blood vessels with evidence of thromboses, which was consistent with the diagnosis of hemangioma. The lesion was completely curetted and autogenous bone was implanted. Pedicle screw fixation was carried out. A drain was left in the incision. The deeper layers, subcutaneous tissue, and skin were then sutured closed. After waking from anesthesia in the recovery room, the patient showed good motion of the extremities.

**Figure 3.**
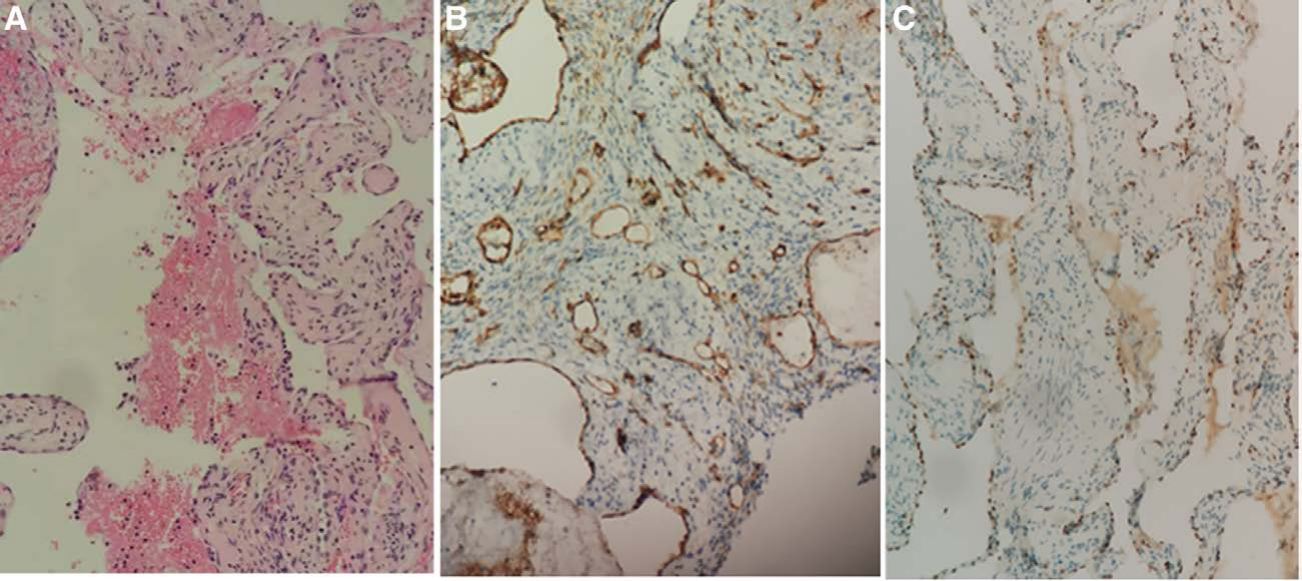
Photomicrographs of hematoxylin and eosin-stained stained sections (200 × magnification) showing the lesion comprises abundant, dilated, proliferated blood vessels containing thromboses (A). Immunohistochemical-stained sections showing positivity for CD31 and ERG (B, C).

On microscopic examination, the lesion comprised abundant, dilated, proliferated blood vessels containing thromboses but contained no adipose or fibrous tissue (Fig. 3A). Immunohistochemical analysis was positive for CD31 and the ETS-1 related gene (Fig. 3B–C).

The patients were in good general condition and free of pain or complications by 9 days after the operation. The incision healed cleanly and she was discharged without complications.

## 3. Discussion

The commonest location of hemangiomas is the spine, specifically the thoracic and lumbar vertebral bodies. The vast majority of VHs remain asymptomatic. They are a frequent, often incidental, finding on CT scans and MRIs of the spine. They can be detected at any age, most often being diagnosed in the fifth decade of life.^[5]^

Perman et al^[6]^ first described the radiographic features of typical VHs in 1926. These include reduced bone density and a coarse, thickened, vertical, trabecular pattern, resembling “honeycomb,” “corduroy cloth,” or “jail bars.” On axial CT images, VHs appear as small, punctate areas of high attenuation, forming a “spotted or “polka-dot appearance. On sagittal and coronal reformatted CT images of VHs, the vertical orientation of the thickened trabeculae resembles “corduroy cloth.”^[7]^

Histological examination of VHs characteristically reveals that they feature thin-walled vessels. Secondary reactive phenomena include small amounts of reactive fibrous tissue and/or adipose involution of bone marrow,^[7]^ the former frequently surrounding the neoplastic vessels. The proportions of blood vessels and adipose tissue differ between lesions. Laredo et al^[4]^ summarized the radiological findings in 32 patients with VHs, all of which had been scored on plain radiographs. Additionally, T1WI and T2WI had been assessed on CT in 19 of those patients, 15 of whom had undergone selective spinal arteriography. In this study, they found that the amount of adipose tissue was inversely related to the number of vessels. The histopathologic diagnosis depends on the appearance of the blood vessels.

VHs show hypo- or hyperintensity on T1WI, depending on the proportions of blood vessels and adipose tissue. Shortened Tl relaxation times reflect the fatty component of these tumors^[8]^; that is, the greater the amount of adipose tissue, the shorter the Tl relaxation time.^[2–4]^ In contrast, the greater the proportion of blood vessels, the longer the T1 relaxation time. VHs show high signal intensity on conventional T2WI MR images, regardless of the proportion of fat and vessels. In the present case, we found hypointensity on T1WI and hyperintensity on T2WI, and histopathological examination revealed a hemangioma that contained no adipose tissue. The findings are consistent with the histopathological findings.

There are different views on the relationship between fatty stroma and the aggressiveness of VHs. Laredo et al^[4]^ found that a higher fat content generally correlates with inertness, whereas when the imaging features suggest a high vascular content, the lesions behave more aggressively, evolving into compressive lesions in 3 patients in their study. Hoyle et al^[3]^ and Friedman^[8]^ found that VHs lacking adipose tissue and with hypointensity on T1WI showed no evidence of aggressive behavior on imaging or during follow-up. Histopathological examination of the present patient VH revealed only blood vessels with no adipose or fibrous tissue and the signal characteristics on T1WI and T2WI images were consistent with this.

VHs can be classified into 3 categories according to the patients’ lesions and symptoms: Enneking Stages 1 (“latent”), mild bony destruction with no symptoms, Enneking Stage II (“active”), bony destruction with pain, Enneking Stage III(“aggressive”), neurological deficit with epidural and/or soft tissue extension.^[9]^ AVHs were uncommon and difficult to diagnosis because the imagine findings were different compared with the typical VHs.Laredo et al^[10]^ described 6 imagines features that were seen significantly more often in aggressive VHs: the entire vertebral body involved; extension into the neural arch; cortical expansion; thoracic location; an irregular honeycomb pattern; a soft tissue mass. Even in the more aggressive lesions, the typical “polka-dot” sign should always be sought on axial CT images.^[7]^ However, there was no “polka-dot” sign on axial CT images and vertical vertebral striation on sagittal reconstruction CT images in present case to make the dilemma in our initial diagnosis. Wang et al^[11]^ summarized the atypical radiographic feature of aggressive VHs, including osteolytic bone destruction, vertebral compression fractures, lesions involving > 1 segment or in which the soft mass extended longitudinally beyond 1 spinal segment, the lesion centered in the pedicle and/or lamina, and atypical MRI signals (e.g., hypointense to isointense) in T2-weighted images. However, even if the lesion involved more than 1 segment or the soft tissue mass extended longitudinally beyond 1 spinal segment, the intervertebral discs were intact according to previous reports. The case we reported was very aggressive, having destroyed multiple vertebral bodies, 2 interverbal discs completely, which form a single cavity. Furthermore, spinal kyphosis formation did not document in previous literature. Therefore, the case we reported was very aggressive and we believe that this lesion aggressive behavior was attributable to its vascular content.

Even the aggressive VHs are benign in nature and rarely progress rapidly, treatment for VHs should base on patients’ symptoms. Reported treatments include radiotherapy, vertebroplasty, direct alcohol injection, embolization, surgery and a combination of these modalities.^[11]^ Even there was no myelopathy in present case, vertebroplasty was performed because of her thoracic kyphosis.

## 4. Conclusion

AVHs destruction multiple vertebrae and interverbal discs and resulting in spinal kyphosis was extremely rare. we believe that this lesion aggressive behavior was attributable to its vascular content.

## Acknowledgments

We thank Dr Trish Reynolds, MBBS, FRACP, from Liwen Bianji (Edanz) (http://www.liwenbianji.cn/), for editing the English text of a draft of this manuscript.

## Author contributions

**Conceptualization:** Chuan-Ping Gao.

**Data curation:** Liang-Jie Wang, Hong-Mei Zou, Guan-Xi Wang.

**Investigation:** Feng-Hou.

**Writing – original draft:** Liang-Jie Wang, Hong-Mei Zou.

**Writing – review & editing:** Chuan-Ping Gao.
